# Image-text guided fundus vessel segmentation via attention mechanism and gated residual learning

**DOI:** 10.3389/fcell.2025.1710343

**Published:** 2025-12-16

**Authors:** Jianguo Xu, Qingyou Liu, Jianxin Shen, Rong Tan, Sukun Tian, Wei Chi, Weihua Yang

**Affiliations:** 1 College of Mechanical and Electrical Engineering, Nanjing University of Aeronautics and Astronautics, Nanjing, China; 2 Center of Digital Dentistry, Peking University School and Hospital of Stomatology & National Engineering Research Center of Oral Biomaterials and Digital Medical Devices & NHC Key Laboratory of Digital Stomatology, Beijing, China; 3 Shenzhen Eye Hospital, Shenzhen Eye Medical Center, Southern Medical University, Shenzhen, China

**Keywords:** fundus vessel segmentation, deep learning, image-text, squeeze-and-excitation, gated residual learning

## Abstract

**Background:**

Fundus vessel segmentation is crucial for the early diagnosis of ocular diseases. However, existing deep learning-based methods, although effective for detecting coarse vessels, still face challenges in segmenting fine vessels and heavily rely on time-consuming and labor-intensive pixel-level annotations.

**Methods:**

To alleviate these limitations, this study proposes an image-text guided segmentation model enhanced with the Squeeze-and-Excitation (SE) module and gated residual learning. Concretely, the multimodal fundus vessel datasets with text labels are primarily constructed, effectively supporting our pioneering effort to successfully introduce an image-text model into fundus vessel segmentation. Secondly, an improved image-text model is meticulously designed, focusing on the following two aspects: (1) embedding the SE module in the CNN backbone to adaptively recalibrate channel weights for enhanced vessel feature representation; (2) integrating gated residual learning into the ViT backbone to dynamically regulate the information flow between image and text features.

**Results:**

Extensive quantitative and qualitative experiments on two publicly available datasets, including DRIVE and ROSE-1, demonstrate that the proposed model achieves superior segmentation performance. Specifically, on the DRIVE dataset, the model attains an F1-score of 82.01%, an accuracy of 95.72%, a sensitivity of 83.25%, and a specificity of 97.43%. On the ROSE-1 dataset, the model records an F1-score of 86.34%, an accuracy of 94.61%, a sensitivity of 90.14%, and a specificity of 95.80%. Compared with most deep learning methods, these results reveal the competitiveness of the improved model, indicating its feasibility and potential value in fundus vessel segmentation, which is expected to expand a new research approach in this field.

## 1 Introduction

**FIGURE 1:**
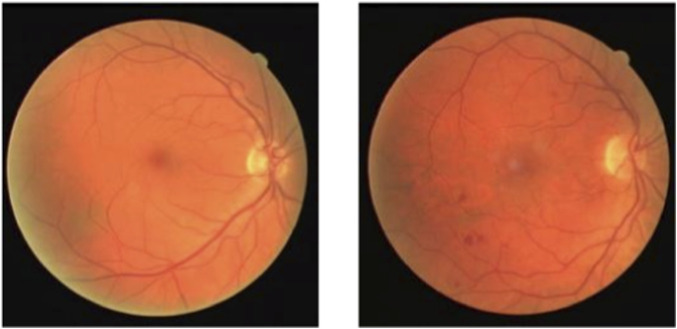
CF images.

With the rapid development of medical imaging technology and equipment, computer-aided medical image analysis has become increasingly important in clinical diagnosis and surgical planning (Tian et al., 2021). Among these, medical image segmentation technology plays a key role in effectively extracting the morphology and spatial information of target areas, which is essential for quantitative analysis (Pham et al., 2000). Color fundus (CF) photography is a commonly used technique for capturing fundus images. A normal CF image is shown in Figure 1(left), which primarily includes the macula, optic disc, retina, and central fundus arteries and veins. In contrast, fundus images of diabetic patients, as shown in Figure 1(right), may exhibit abnormal vessel end-stage bleeding, where unclear boundaries and irregular dark red areas appear (Grinsven et al., 2016). Patients with hypertension and coronary heart disease may show fundus arterial sclerosis, while glaucoma patients may exhibit vessel changes in the optic disc (Dervisevic et al., 2016). Therefore, accurate segmentation of vessels in CF images is of great importance in assisting the early diagnosis of related diseases.

**FIGURE 2:**
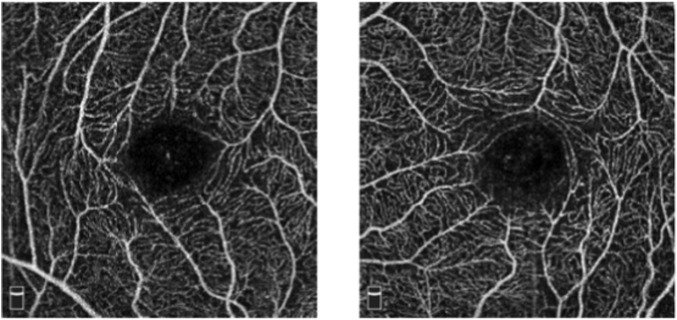
OCTA images.

However, CF photography only captures coarse vessels and often fails to image microvessels in the foveal region. Fluorescein angiography and indocyanine green angiography can differentiate fundus vessel systems, including capillaries, but they are invasive techniques that require intravenous imaging medium. Additionally, allergic reactions and serious side effects may occur (Witmer et al., 2013). In contrast, Optical Coherence Tomography Angiography (OCTA) is an emerging non-invasive imaging technology developed further from optical coherence tomography (OCT) (Lee et al., 2017; Li et al., 2021), which can generate high-resolution 3D images of the fundus vessel system (As shown in Figure 2). and has increasingly been accepted as a tool for observing vessels (Leitgeb, 2019). Research has shown that abnormalities in fundus microvessels, as revealed by OCTA imaging, can often provide early indications of certain systemic diseases, offering significant value in guiding disease diagnosis and treatment. For example, in patients with glaucomatous optic neuropathy, OCTA images show significantly lower blood flow density in the optic disc, peripapillary, and macular regions compared to healthy eyes (Alnawaiseh et al., 2018). Fundus microvessels in conditions such as diabetic retinopathy and age-related macular degeneration also undergo pathological changes (Zhao et al., 2017). Recent studies have also indicated that changes in the microvessel morphology displayed on OCTA images are associated with Alzheimer’s disease and mild cognitive impairment (Yoon et al., 2019). Hence, the automatic detection of fundus vessel in OCTA images are of crucial value for the early diagnosis of fundus diseases.

Traditional vessel segmentation methods include line detectors (Ricci and Perfetti, 2007), multi-scale filtering (Li et al., 2012), and particle swarm optimization algorithms (Sreejini and Govindan, 2015), which require high-quality image and are significantly influenced by the fundus structure. In recent years, deep learning demonstrated outstanding performance in medical image segmentation (Jian et al., 2025; Shi et al., 2025; Yu et al., 2026; Aljohani, 2025; Iqbal et al., 2024). The introduction of U-Net (Ronneberger et al., 2015) popularized U-shaped networks, and many improved fundus vessel segmentation models were developed based on this architecture. LadderNet (Zhuang, 2019), which incorporated multiple encoder-decoder pairs and used shared-weight residual blocks. CE-Net (Gu et al., 2019) enhanced boundary accuracy and segmentation clarity by adding a context encoding module, particularly improving vessel boundary identification in complex backgrounds. CS-Net (Mou et al., 2019) improved U-Net with attention modules and 1 × 3/3 × 1 convolutions to improve segmentation. R2UNet (Alom et al., 2019) featured recursive residual convolution layers that accumulated feature information. This design ensured better feature representation for segmentation tasks. DenseUNet (Cao et al., 2020) employed a weighted loss function to enhance vessel segmentation performance. Attention U-Net (Oktay et al., 2018) introduced a novel attention gate that eliminated the need for explicit external localization modules when using cascaded convolutional neural networks.

Additionally, Lin et al. (2021) combined the advantages of the hierarchical Swin Transformer with the standard U-Net model to improve semantic segmentation quality for various medical images. Chen et al. (2021) proposed TransUNet (Transformer and U-Net), which combined the strengths of Transformer and U-Net and leveraged the global context-capturing ability of Transformer and the fine feature extraction capability of U-Net. TransUNet fully leveraged the global context-capturing ability of Transformer and the fine feature extraction capability of U-Net, opening new research directions in the field of medical image segmentation. Yang and Tian (2022) offered an improved version, TransNUNet, which enhanced feature selection during the upsampling phase by introducing a convolutional attention module, thereby improving segmentation accuracy. Wang et al. (2023) introduced Cross-Convolution Transformer, which improved the segmentation quality of target boundary contours by combining a multi-scale edge feature fusion module.

From the above analysis, it is evident that deep learning-based segmentation methods have become the mainstream approach for addressing fundus vessel detection task. They have demonstrated significant potential in solving the challenges associated with manual vessel segmentation in clinical practice and offer better applicability compared to traditional image processing solutions. However, while these methods typically perform well in coarse vessel detection tasks, there is still room for improvement in segmenting fine vessels. Additionally, deep learning-based vessel segmentation methods heavily rely on pixel-level annotated data, which requires labor-intensive and time-consuming labeling processes, and their performance tends to degrade under small-sample condition. Therefore, this paper proposes a novel method, namely Image-Text Guided Fundus Vessel Segmentation via Attention Mechanism and Gated Residual Learning, which aims to reduce dependency on pixel-level annotations by jointly leveraging image and textual information, and enhances fine vessel capture through attention mechanism and gated residual learning.

The main contributions are as follows.

1. The multimodal fundus vessel datasets with text labels are constructed, which play a crucial role in the multimodal representation of vessel information and pave the way for exploring the integration of textual and image features in the vessel segmentation task.
2. Based on the constructed multimodal datasets, an existing image-text model is successfully adapted to the vessel segmentation task, thereby validating the feasibility of leveraging textual information to enhance segmentation performance.
3. An improved image-text model is further designed, where the SE module enhances channel-wise feature representation by adaptively recalibrating channel weights, while gated residual learning improves both stability and performance by dynamically regulating information flow.
4. Extensive quantitative and qualitative experiments are carried out on two types of datasets (i.e., the CF dataset and the OCTA dataset), and our method achieves significant gains in vessel segmentation, highlighting its potential for clinical applications.

The remaining structure of this paper is organized as follows: Section 2 reviews related work. Section 3 elaborates on the implementation details of the proposed methodology. Section 4 presents experimental results and discussions. Section 5 concludes the study.

## 2 Related work

### 2.1 LViT architecture

The LViT (Li et al., 2024) model consists of a U-shaped CNN branch and a U-shaped ViT branch. The CNN branch serves as the information input source, with its segmentation head generating the predicted masks. The ViT branch fuses image and textual information, leveraging the Transformer’s capability to process cross-modal data. Specifically, textual data is first simplified and vectorized, then combined with image vectors, which are passed into the U-shaped ViT branch for processing. Additionally, a Pixel-Level Attention Module (PLAM) is inserted at the skip connections in the U-shaped CNN branch, enabling LViT to maintain local image features while integrating more textual semantic information.

### 2.2 BERT language model

BERT (Bidirectional Encoder Representations from Transformers) is a pre-trained language model proposed by Google AI (Devlin et al., 2019) that has achieved remarkable performance on top-level tasks such as the SQuAD1.1 machine reading comprehension benchmark. The innovation of BERT lies in its bidirectional encoder architecture, which teaches word semantics simultaneously from both the left and right contexts. Unlike traditional sequence-based language models (e.g., LSTM and GRU), BERT is based on the Transformer architecture, which processes all input data in parallel and efficiently captures long-range dependencies.

### 2.3 Pixel-level attention module

**FIGURE 3:**
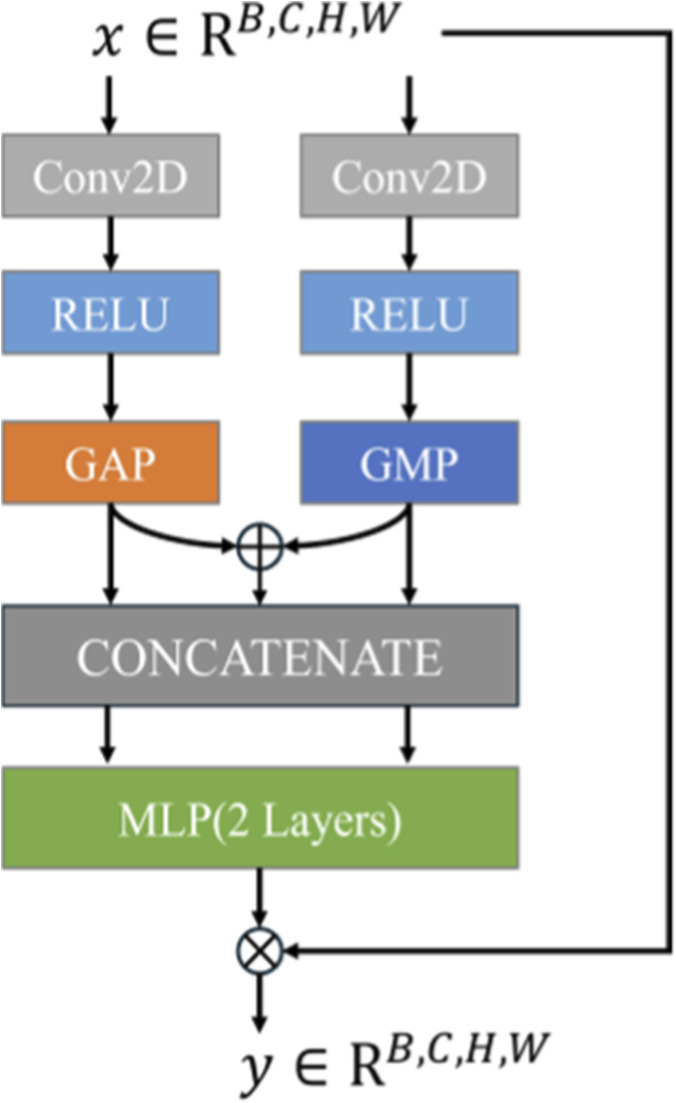
PLAM structure.

PLAM (Figure 3) is designed to preserve local image features while further integrating semantic features from the text. Inspired by CBAM (Convolutional Block Attention Module) (Woo et al., 2018), it employs parallel branches to perform Global Average Pooling (GAP) and Global Max Pooling (GMP) operations. PLAM significantly enhances medical image segmentation performance by addressing key limitations of Transformer-based models. Specifically, PLAM strengthens local features, counteracting the tendency of Transformer models to prioritize global features, and incorporates both channel attention and spatial attention mechanisms.

## 3 The proposed methods

In this part, we present the improved image-text guided fundus vessel segmentation model. First, the optimal placement of the SE module within the model is explored, focusing on its integration after downsampling, upsampling, or all convolutional layers to maximize performance. Next, we raise a gated residual learning-based transformer layer that adaptively controls information flow in the ViT branch, thereby improving model flexibility and stability. Additionally, the process of generating text labels is outlined, highlighting their role in guiding the segmentation task. Finally, the structure of the proposed model is illustrated, demonstrating how these components are integrated to enhance performance in fundus vessel segmentation.

### 3.1 Position design of SE module

**FIGURE 4:**
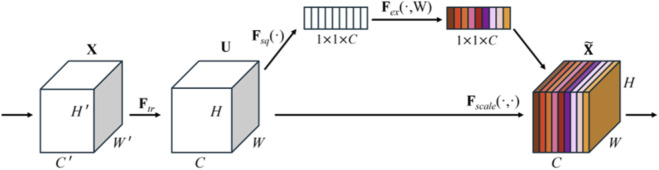
SE module structure.

The SE module (Hu et al., 2020) is a channel attention mechanism that adaptively weights feature channels through three key steps: Squeeze, Excitation, and Recalibration. These steps dynamically adjust the responses of each channel in the feature maps, enabling the network to focus more on channels that contribute to the task while suppressing irrelevant ones. The process of the SE module is illustrated in Figure 4.

Before feature enhancement, the input feature $X\in R^{H^{\prime }\times W^{\prime }\times C^{\prime }}$ is transformed, where $H^{\prime }$ and $W^{\prime }$ represent the height and width of the feature map, and $C^{\prime }$ is the number of channels. After $F_{tr}$ the feature map X becomes the feature map U . $F_{tr}$ is a standard convolution operator. This process can be expressed by Equation 1:

$$U=V_{c}\cdot X=\sum_{s=1}^{C^{\prime }}V_{c}^{s}\cdot X^{s}$$ (1)

where, $V_{c}$ refers to the parameter of the *c-*th convolution kernel, $V_{c}^{s}$ represents a 2D spatial kernel, and $X^{s}$ denotes the *s*-th input.

The squeeze step can be expressed as Equation 2:

$$z_{c}=F_{sq}\left(u_{c}\right)=\frac{1}{H\times W}\sum_{i=1}^{H}\sum_{j=1}^{W}u_{ijc}$$ (2)

where $z_{c}$ is the global descriptor for the *c*-th channel, and $u_{ijc}$ represents the value at position $\left(i,j\right)$ in the *c*-th channel of the input feature map U .

The excitation step is mathematically written as Equation 3:

$$s=F_{ex}\left(z,W\right)=\sigma \left(W_{2}\cdot \text{ReLU}\left(W_{1}\cdot z\right)\right)$$ (3)

where $W_{1}$ and $W_{2}$ are the weight matrices of the two fully connected layers, and σ represents the Sigmoid function.

The recalibration step can be formalized as Equation 4:

$${\tilde{X}}_{c}=F_{scale}\left(u_{c},s_{c}\right)=s_{c}\times u_{c}$$ (4)

where $u_{c}$ is the *c*-th channel in the input feature map, $s_{c}$ is the excitation value for that channel, and ${\tilde{X}}_{c}$ is the adjusted feature map.

**FIGURE 5:**
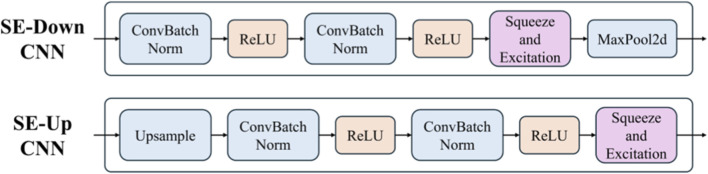
The SE module position. The upper row is added in the downsampling stage, and the bottom row is added in the upsampling stage.

As an adaptive channel attention mechanism, the SE module can effectively adjust the importance of feature map channels, which strengthens the network’s sensitivity to key features. However, when adding the SE module to the LViT model, choosing the appropriate insertion position is crucial. This subsection investigates the advantages and disadvantages of inserting the SE module after the downsampling convolution layers, upsampling convolution layers, and all convolution layers, offering theoretical support for subsequent experiments. The different structures are shown in Figure 5.

#### 3.1.1 After downsampling convolution layers

The downsampling layer reduces the resolution of the feature map and enhances deep semantic information. Inserting the SE module at this position can effectively strengthen the expression of low-level features, thus preventing the loss of important details and helping capture basic features like edges and textures. Since the computational cost is lower, this design alleviates the network’s burden. However, over-reliance on low-level features may degrade performance in more complex tasks.

#### 3.1.2 After upsampling convolution layer

The upsampling layer recovers the spatial resolution of the feature map and strengthens high-level semantic information. Adding the SE module after this layer can strengthen the weight of high-level features, which makes the network more precise in recovering details. This adjustment benefits fine-grained tasks. However, excessive weighting of high-level features may lead to overfitting, especially with limited data, which affects the model’s generalization ability.

#### 3.1.3 After all convolution layers

Adding the SE module after all convolution layers strengthens the expression of features at all levels, making it particularly suitable for tasks requiring multi-scale feature fusion. While this design improves feature representation by integrating low- and high-level information, it may result in excessive weighting of low-level features, potentially distorting high-level semantic information and degrading information transmission.

The specific placement of the SE module will be further discussed in the experimental section to determine the optimal configuration based on the results.

### 3.2 Transformer layer based on gated residual learning

Gated residual learning (Chen et al., 2017) combines residual connections with a gated mechanism. Unlike traditional residual connections, gated residual learning dynamically computes a gated signal for the input features, controlling the degree of weight in the residual connection. This enables the network to adaptively adjust the residual signal at each layer, which enhances the model’s flexibility and adaptability. The process is expressed as Equation 5:

$$y=\text{Gate}\left(x\right)\cdot F\left(x\right)+x$$ (5)

where x is the input, $F\left(x\right)$ is the output after passing through several layers of the network, and $\text{Gate}\left(x\right)$ is the gated signal, controlling the strength of the information flow. The gated signal is generated through a fully connected layer and Sigmoid function. The final output is y .

**FIGURE 6:**
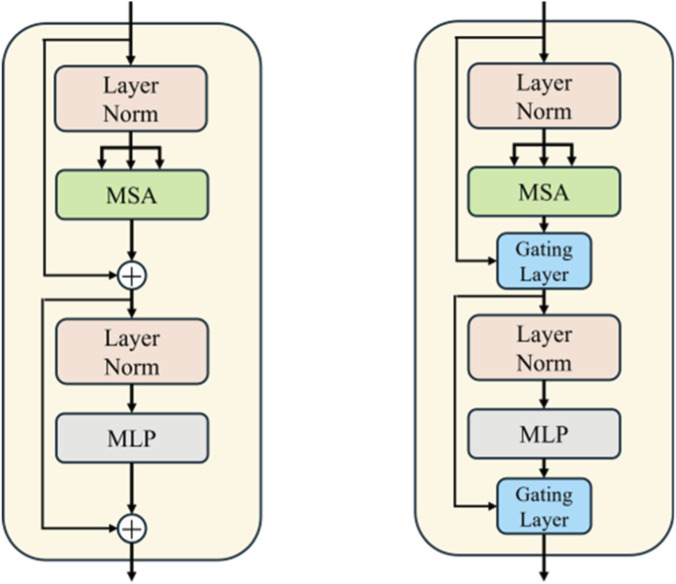
Gated residual learning diagram. The left structure is the original transformer layer, while the right structure is the transformer layer with gated residual learning.

Taking inspiration from the above, we innovatively integrated the gated residual learning into the ViT branch to enhance its expressive power and training stability. By adding a learnable gated signal to each layer’s output, the model can more flexibly control the flow of information, which boosts the performance of the vessel segmentation task. The gated residual learning is applied to the residual connections in both the self-attention layer and the feed-forward network layer. Figure 6 shows a schematic of gated residual learning, which illustrates how the gated mechanism adjusts the information flow in the self-attention and feed-forward network layers.

In the self-attention and feed-forward network parts, the weighted sum of the output y and input x is adjusted by the gated signal, as shown in Equations 6, 7.

$$y^{\prime }={\text{Gate}}_{\text{attn}}\left(x\right)\cdot \text{Attn}\left(x\right)+x$$ (6)

$$y={\text{Gate}}_{\text{mlp}}\left(y^{\prime }\right)\cdot \text{MLP}\left(y^{\prime }\right)+y^{\prime }$$ (7)

The effectiveness of gated residual learning has been confirmed in many works, including reference (Chen et al., 2017). In this study, we directly integrate this module into the ViT branch of the improved image-text model.

### 3.3 Text label design

Since the CF and OCTA datasets lack text labels for vessel segmentation, we generate image-specific text labels by combining descriptive texts related to the fundus vessels, thereby preparing for subsequent multimodal feature fusion. The process is detailed as follows.

- First, the fundus images in the dataset undergo preprocessing, including grayscale conversion, normalization, CLAHE (Contrast Limited Adaptive Histogram Equalization), and gamma correction, to ensure consistency when integrating textual information in subsequent steps.
- Then, based on the content of the images and incorporating features of the fundus vessels, relevant textual descriptions are generated. The text generation strategy employed in this process is primarily based on three aspects: vessel density, vessel distribution, and abnormal areas, with the vessel features described in natural language under the guidance of ophthalmologists. For example, a description for one image might include: “The vessels radiate from the periphery to the center in a root-like pattern, gradually thinning toward the center, leaving the middle of the image free of vessels.” These textual descriptions contain not only the vessel features in the image but also key semantic information that may impact the segmentation task.
- Finally, the text labels are aligned with the image data and formatted for storage, facilitating subsequent vessel segmentation task. The labels are stored in an EXCEL file, where each label is associated with its corresponding image file name to ensure accurate matching of label information. During the label generation process, to ensure clarity and consistency of the semantics, medical expert knowledge is consulted, thereby guaranteeing the accuracy of the descriptive content.

**FIGURE 7:**
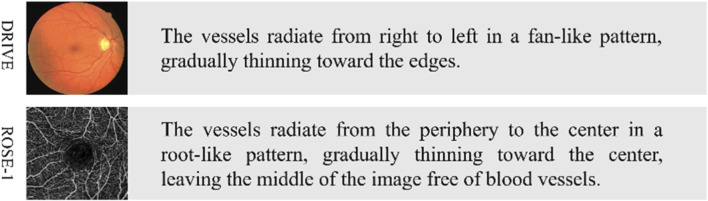
Example of text labels. The left side shows the original images from the CF and OCTA datasets, while the right side displays their corresponding text labels.

Through the above process, we have established text labels corresponding to the images from the DRIVE and ROSE-1 datasets, laying the foundation for subsequent exploration of the performance of the proposed model. Specific examples are illustrated in Figure 7.

### 3.4 Structure of the improved image-text model

**FIGURE 8:**
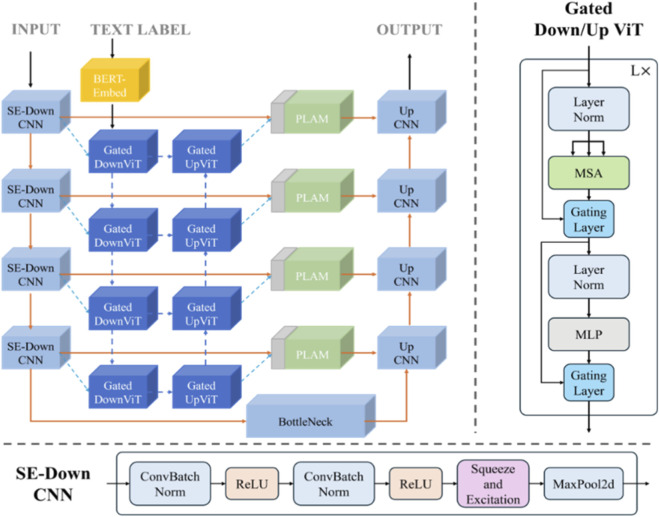
The improved image-text model structure.

Building upon the aforementioned foundation, the improved image-text model structure (Figure 8) is formally designed, in which the SE module is integrated into the downsampling stage of the CNN branch to enhance feature representation, and gated residual learning is applied throughout the ViT branch to facilitate effective information flow and cross-modal feature fusion. Additionally, the feature extraction and fusion process for text and images remains consistent with LViT. Specifically, a pre-trained BERT model is employed for text feature extraction, a CNN model is used for image feature extraction, and the heterogeneous features are subsequently fused within the ViT branch.

## 4 Results and discussions

### 4.1 Datasets

In this study, two public datasets are used: the CF dataset (i.e., DRIVE (Staal et al., 2004)) and the OCTA dataset (i.e., ROSE-1 (Ma et al., 2020)). These datasets contain a variety of fundus vessel images and their corresponding ground truth images, which offer rich data resources for research. The DRIVE dataset includes 40 fundus vessel images with a resolution of 584 × 564 pixels, where the image resolution is 584 × 564 pixels. The dataset has been divided into a training set and a testing set by the official source, with 20 images for training and 20 for testing. The ROSE-1 dataset contains 39 superficial OCTA fundus images with a resolution of 304 × 304 pixels. Of these images, 30 are allocated to the training set and 9 to the testing set.

The proposed model was trained for 300 epochs with a batch size of 2, using the Adam optimizer and a learning rate of 0.001, and implemented in Python using PyTorch for training and testing, with an NVIDIA RTX 4080 GPU for computation.

### 4.2 Evaluation metrics

To evaluate the segmentation performance of the proposed model, we performed qualitative and quantitative analysis. Qualitative analysis involves comparing the vessel segmentation results to assess segmentation quality. Quantitative analysis directly compares the values of the following evaluation metrics: F1-score, Accuracy (ACC), Sensitivity (SE) and Specificity (SP). F1-score measures the similarity between the segmentation results and the ground truth. Accuracy indicates the proportion of correctly segmented pixels in the entire image. Sensitivity indicates the proportion of correctly segmented vessel pixels. Specificity indicates the proportion of correctly segmented background pixels. These metrics are calculated using Equations 8–11:

$$F1=\frac{2\text{TP}}{2\text{TP}+\text{FP}+\text{FN}}$$ (8)

$$\text{Acc}=\frac{\text{TP}+\text{TN}}{\text{TP}+\text{TN}+\text{FP}+\text{FN}}$$ (9)

$$\text{Sp}=\frac{\text{TN}}{\text{TN}+\text{FP}}$$ (10)

$$\text{Se}=\frac{\text{TP}}{\text{TP}+\text{FN}}$$ (11)

where TP (True Positive) represents pixels correctly predicted as vessel, TN (True Negative) represents pixels correctly predicted as non-vessel, FP (False Positive) represents pixels incorrectly predicted as vessel, and FN (False Negative) represents pixels incorrectly predicted as non-vessel.

### 4.3 Discussion on experimental results of CF dataset

#### 4.3.1 Discussion on different adding positions of SE module

The purpose of this experiment is to explore the effect of adding SE module at different positions in the CNN branch of the LViT model on the model segmentation performance. The DRIVE dataset is utilized for experiments. The specific experimental models include the following.

- BM: The baseline model (i.e., LViT) without the SE module.
- SE-DOWN: The SE module is added only after the downsampling convolution layers.
- SE-UP: The SE module is added only after the upsampling convolution layers.
- SE-ALL: The SE module is added after every convolution layer.

##### 4.3.1.1 Qualitative analysis

**FIGURE 9:**
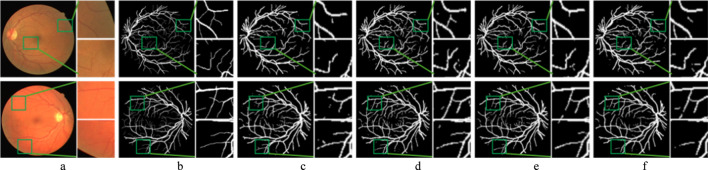
Segmentation results of the improved model with SE module in different positions. Each image is followed by two locally enlarged images. **(a)** CF image. **(b)** Ground truth. **(c)** BM. **(d)** SE-DOWN. **(e)** SE-UP. **(f)** SE-ALL.

Figure 9 presents the comparison of the segmentation results of adding SE module at different positions of the CNN branch. The BM performs poorly in vessel segmentation, mainly due to vessel edge fragmentation and missed detections. In contrast, the model with the SE-DOWN improves vessel continuity and detail representation, effectively suppressing background noise and enhancing segmentation accuracy. The SE-UP shows some improvement in vessel detail recovery but still exhibits slight over-segmentation or blurry details. However, the SE-ALL performs even worse than the BM, which results in noticeable under-segmentation and mis-segmentation, particularly in complex vessel regions.

The reasons for these results have been preliminarily discussed in the previous section. The detailed information of vessels is often an important representation of deep features. Therefore, adding the SE module during the downsampling phase allows for better capture of these details, thereby boosting the accuracy of vessel segmentation. The upsampling phase mainly focuses on feature recovery and detail reconstruction, where shallow features are more critical. Although the SE module can enhance the expression of important features, its impact is relatively small during this phase, as the model has already extracted sufficient features during the downsampling phase. Consequently, adding the SE module during the upsampling phase may slightly optimize features, but its effect is more limited compared to the downsampling phase. Furthermore, adding the SE module at all stages leads to excessive features of weighting and suppression. In some less important convolution layers, the SE module may suppress shallow feature information that is useful for vessel segmentation, which affects the model’s performance and results in less accurate feature extraction.

##### 4.3.1.2 Quantitative analysis

Table 1 summarizes the quantitative results when the SE module is placed at different positions, which provides detailed insights into the impact of SE module placement on model performance. Bold values in the table indicate the maximum value for this evaluation metric. The meaning of the bold values in subsequent tables remains the same. With an F1-score of 82.14%, the SE-DOWN model outperforms the BM by 1.30%, demonstrating its superior performance in the vessel segmentation task. Furthermore, the SE-DOWN model also excels in Se, reaching a score of 81.86%, which represents a 3.15% increase over BM. In terms of Acc and Sp, the SE-DOWN model shows only slight differences from the BM, with a difference of 0.20% and −0.19%, respectively. These results indicate that the SE-DOWN model maintains a stable ability to balance positive and negative sample recognition while preserving segmentation accuracy. In comparison, although the SE-UP and SE-ALL models also show gains in F1-score and Se, the enhancements are relatively smaller. Therefore, based on both qualitative and quantitative analyses, this study ultimately decides to add the SE module after the convolutional layer in the downsampling stage.

**TABLE 1:** Comparison of evaluation metrics of SE module in different positions.

Model	F1-score	Acc	Se	Sp
BM	80.84%	95.62%	78.71%	**97.93%**
SE-DOWN	**82.14%**	**95.82%**	**81.86%**	97.74%
SE-UP	81.16%	95.60%	80.72%	97.64%
SE-ALL	81.12%	95.64%	79.74%	97.81%

Bold values in the table indicate the maximum value for this evaluation metric. The meaning of the bold values in subsequent tables remains the same.

#### 4.3.2 Discussion on segmentation performance of different models

##### 4.3.2.1 Qualitative analysis

**FIGURE 10:**
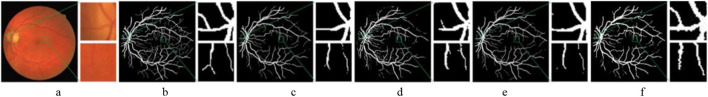
Comparison of segmentation results of different models. **(a)** CF image. **(b)** Ground truth. **(c)** U-Net. **(d)** R2U-Net. **(e)** Attention U-Net. **(f)** Ours.

Figure 10 shows that the original U-Net model produces suboptimal segmentation results. On the DRIVE dataset, the location of large vessels is often inaccurately identified, which leads to a significant number of misclassifications. R2U-Net, by utilizing recursive convolution structures to accumulate features, captures more detailed information. However, this detail often appears fragmented, resulting in poor continuity. Attention U-Net addresses this issue by incorporating a gated attention mechanism, which effectively enhances the propagation of vessel information while suppressing irrelevant features. Compared to U-Net and R2U-Net, it achieves better segmentation performance. The proposed model (Ours) outperforms Attention U-Net in vessel segmentation, capturing more detailed fine vessel features with improved continuity and achieving more accurate segmentation for coarse vessels.

##### 4.3.2.2 Quantitative analysis

As shown in Table 2, in the experiment on the DRIVE dataset, the proposed model achieves the best Se, improving by 3.40% compared to the second-best model. Overall, it demonstrates a significant advantage over models such as U-Net and R2U-Net. At a 50% sample rate, the performance of the proposed model is very close to that of ResDO-UNet. Although it slightly lags in terms of F1-score, the proposed model shows a significant advantage in Se, reaching 83.16%, outperforming most other models. This indicates that the proposed model rarely misclassifies actual vessel regions as non-vessel areas. Furthermore, comparing models with and without text labels highlights the importance of text labels in the vessel segmentation task. Models without text labels generally perform worse than those with text labels at the same sample rate. Specifically, at a 25% sample rate, the use of text labels improves the model’s evaluation metrics by 2.74%, 0.60%, 4.14%, and 0.14%, respectively.

**TABLE 2:** Comparison of different models on the DRIVE dataset.

Model	Text label	Sample rates	F1-score	Acc	Se	Sp
U-Net (Ronneberger et al., 2015)	×	100%	81.42%	95.31%	75.37%	98.20%
LadderNet (Zhuang, 2019)	×	100%	82.02%	95.61%	78.56%	98.10%
R2U-Net (Alom et al., 2019)	×	100%	81.71%	95.56%	77.92%	98.13%
DenseUNet (Cao et al., 2020)	×	100%	80.86%	95.28%	78.30%	97.75%
Attention U-Net (Oktay et al., 2018)	×	100%	80.37%	**96.10%**	79.05%	98.31%
IterNet (Li et al., 2020)	×	100%	82.05%	95.73%	77.35%	**98.38%**
ResDO-UNet (Liu et al., 2023)	×	100%	**82.29%**	95.61%	79.85%	97.91%
Ours	×	25%	76.97%	94.48%	78.24%	96.68%
	√	25%	79.71%	95.08%	82.38%	96.82%
	×	50%	81.24%	95.50%	83.07%	97.20%
	√	50%	81.30%	95.51%	83.16%	97.21%
	×	100%	80.67%	95.65%	77.31%	98.14%
	√	100%	82.01%	95.72%	**83.25%**	97.43%

Bold values in the table indicate the maximum value for this evaluation metric. The meaning of the bold values in subsequent tables remains the same.

Overall, the advantage of the proposed model lies not only in a single metric but in achieving a balanced improvement across multiple key evaluation metrics. Especially under low sample rate conditions, the introduction of text labels significantly enhances the accuracy of vessel segmentation. Moreover, the proposed model demonstrates strong competitiveness in both ACC and Se, effectively improving the accuracy and robustness of the segmentation results.

### 4.4 Discussion on experimental results of OCTA dataset

To further explore the improved model’s performance in different image modalities, this study further evaluates the proposed model on the OCTA dataset (i.e., ROSE-1). In this part, the model naming strategy is consistent with the experiments based on the CF dataset.

#### 4.4.1 Discussion on different adding positions of SE module

##### 4.4.1.1 Qualitative analysis

**FIGURE 11:**
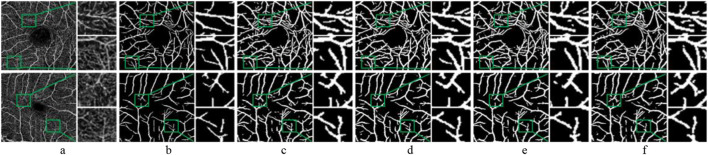
Segmentation results of the improved model with SE module in different positions. Each image is followed by two locally enlarged images. **(a)** OCTA image. **(b)** Ground truth. **(c)** BM. **(d)** SE-DOWN. **(e)** SE-UP. **(f)** SE-ALL.

As shown in Figure 11, the experimental results on the OCTA dataset are like those on the CF dataset. The BM model still performs poorly on the OCTA dataset, with noticeable issues in vessel detection, particularly in the missed segments. The SE-DOWN configuration significantly enhances vessel continuity and detail representation. Although SE-UP and SE-ALL show improvements in some fine details, they still tend to lead to over-segmentation when processing OCTA images. Overall, the SE-DOWN configuration exhibits consistent performance on the OCTA dataset, like its results in the CF modality. This demonstrates its adaptability and potential advantages in multimodal vessel segmentation task.

##### 4.4.1.2 Quantitative analysis

Table 3 further demonstrates that the segmentation performance of SE-DOWN surpasses other models, aligning closely with the experimental results on the CF dataset. The F1-score of SE-DOWN reaches 86.76%. Although it does not achieve the highest values for Acc, Se, and Sp, the results are nearly identical to the optimal values. Through both qualitative and quantitative analysis, it is evident that incorporating the SE module after the downsampling convolution operation is an effective strategy, enhancing the model’s segmentation performance.

**TABLE 3:** Comparison of evaluation metrics of SE module in different positions.

Model	F1-score	Acc	Se	Sp
BM	86.21%	94.38%	89.25%	**95.61%**
SE-DOWN	**86.76%**	94.39%	89.70%	95.45%
SE-UP	86.26%	94.35%	**90.11%**	95.38%
SE-ALL	86.42%	**94.45%**	89.65%	95.61%

Bold values in the table indicate the maximum value for this evaluation metric. The meaning of the bold values in subsequent tables remains the same.

#### 4.4.2 Discussion on segmentation performance of different models

##### 4.4.2.1 Qualitative analysis

**FIGURE 12:**
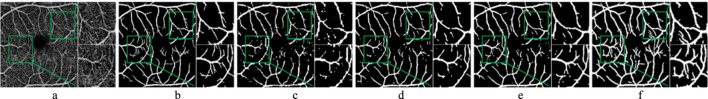
Comparison of segmentation results of different models. **(a)** OCTA image. **(b)** Ground truth. **(c)** U-Net. **(d)** CE-Net. **(e)** CS-Net. **(f)** Ours.

By examining the segmentation results in Figure 12, it is evident that the first three comparison models exhibit a considerable number of white spots, which indicates that the segmented fundus vessels contain more discontinuities and exhibit poor vessel continuity. This issue likely stems from their limited ability to capture fine vessels and handle complex background noise. In contrast, CS-Net displays better vessel continuity, which can be attributed to its focus on the elongated tubular structure of vessels and its network design incorporating prior knowledge. This design enables CS-Net to better preserve the topological structure of vessels, resulting in smoother and more connected segmentation results.

However, upon further inspection of the zoomed-in area, the proposed model shows fewer vessel discontinuities and clearer segmentation at the vessel endpoints. This improvement can be attributed to the integration of the SE module and gated residual learning, which enhances the model’s ability to capture subtle vessel features and maintain structural integrity. Specifically, the SE module strengthens the representation of critical vessel patterns, while the gated residual learning facilitates more effective information flow, enabling the model to better handle challenging cases such as fine vessel endpoints and complex bifurcations. These advantages highlight the proposed model’s superior performance in preserving vessel continuity and accurately segmenting intricate vessels.

##### 4.4.2.2 Quantitative analysis

The data in Table 4 further demonstrate that the proposed model outperforms the other listed models in terms of overall performance. Specifically, the proposed model achieves a 7.92% improvement in F1-score and a 2.14% improvement in Acc compared to the second-best model, even with only 25% of the samples. These results highlight the model’s ability to maintain high segmentation accuracy under low sample rate conditions, which is particularly valuable in scenarios where pixel-level annotated samples are limited.

**TABLE 4:** Comparison of different models on the ROSE-1 dataset.

Model	Text label	Sample rates	F1-score	Acc	Se	Sp
U-Net (Ronneberger et al., 2015)	×	100%	71.16%	89.55%	81.25%	**97.83%**
CE-Net (Gu et al., 2019)	×	100%	75.11%	91.21%	**91.67%**	96.05%
CS-Net (Mou et al., 2019)	×	100%	76.08%	91.52%	86.41%	97.54%
OCTA-Net (Ma et al., 2020)	×	100%	76.97%	91.82%	---	---
Three-Stage (Yan et al., 2018)	×	100%	76.63%	91.79%	---	---
DUNet (Jin et al., 2019)	×	100%	75.05%	91.18%	---	---
Ours	×	25%	84.10%	93.36%	88.96%	94.40%
	√	25%	84.89%	93.96%	86.44%	95.76%
	×	50%	85.49%	94.06%	88.98%	95.27%
	√	50%	85.99%	94.24%	89.73%	95.32%
	×	100%	86.24%	94.34%	90.21%	95.36%
	√	100%	**86.34%**	**94.61%**	90.14%	95.80%

Bold values in the table indicate the maximum value for this evaluation metric. The meaning of the bold values in subsequent tables remains the same.

The experiments conducted under different sample rates, along with the analysis of the impact of text labels, validate the significant role of textual information in guiding the segmentation task. When text labels are incorporated, the model generally outperforms its counterparts without text labels. For instance, at a 25% sample rate, the use of text labels improves the model’s F1-score by 0.79% and Sp by 1.36%, demonstrating the effectiveness of textual priors in enhancing the model’s ability to capture fine vessels and reduce segmentation errors.

### 4.5 Limitations and future work

The proposed model demonstrates significant advantages in the fundus vessel segmentation task, particularly in capturing fine vessels and improving segmentation accuracy under the low sample rate condition. However, several limitations remain in the current study. First, while the model demonstrates competitive performance on public datasets (i.e., DRIVE and ROSE-1), its ability to accurately segment fine vessels still needs to be strengthened in images with severe pathology. Second, the design of text labels relies on manual effort, and the descriptions of vessel morphology remain relatively simplistic, which may limit their effectiveness in guiding the segmentation task. In future work, we plan to address these limitations through the following directions. (1) Fine Vessel Segmentation: Enhance the model’s ability to accurately segment fine vessels by exploring advanced attention mechanisms and multi-scale feature fusion techniques. This will focus on improving the representation of subtle vessels and reducing segmentation errors in complex regions. (2) Automated Text Label Generation: Develop automated or semi-automated methods for generating high-quality textual descriptions by integrating large language models (LLMs) with domain-specific clinical knowledge in ophthalmology. This will enable more detailed and accurate descriptions of vessel morphology, reducing dependency on manual annotation and improving consistency.

## 5 Conclusion

To alleviate the challenges faced by existing deep learning methods in the fundus vessel segmentation task, this study proposes an improved image-text guided fundus vessel segmentation model. On the one hand, we construct the first multimodal fundus vessel segmentation datasets with text labels, providing a valuable resource for subsequent research. On the other hand, after successfully integrating an image-text model into the fundus vessel segmentation scenario, we propose an improved image-text model jointly driven by both the SE module and gated residual learning. This enhancement boosts vessel segmentation performance by refining vessel feature representation and dynamically regulating multimodal information flow. Quantitative and qualitative experiments on both CF and OCTA datasets demonstrate the model’s competitive advantages in the vessel segmentation task. This exploration provides a new path to further enhance the performance of deep learning methods in fundus vessel segmentation while reducing the need for pixel-level annotated samples.

## Data Availability

The original contributions presented in the study are included in the article/supplementary material, further inquiries can be directed to the corresponding authors.
